# An Assessment of Retinal Nerve Fiber Layer Thickness in Non-Diabetic Obese Children and Adolescents

**DOI:** 10.4274/jcrpe.4810

**Published:** 2018-02-26

**Authors:** Bediz Özen, Hakan Öztürk, Gönül Çatlı, Bumin Dündar

**Affiliations:** 1 University of Health Sciences, Tepecik Training and Research Hospital, Clinic of Ophthalmology, İzmir, Turkey; 2 Katip Çelebi University Faculty of Medicine, Department of Pediatric Endocrinology, İzmir, Turkey

**Keywords:** Obesity, optical coherence tomography, children, retinal nerve fiber layer

## Abstract

**Objective:**

Obesity affects almost all systems in the body. This includes the retinal nerve fibers which may be damaged due to a chronic inflammatory process. To determine changes in retinal nerve fiber layer (RNFL) thickness in non-diabetic children and adolescents using optical coherence tomography (OCT) and to evaluate the relationship between this change, metabolic risk factors and pubertal stage.

**Methods:**

Thirty-eight obese and 40 healthy children and adolescents aged 10-18 years were included in the study. RNFL measurements from the optic disk and all surrounding quadrants were obtained using OCT from both eyes of the individuals in the study groups. Correlations between RNFL thickness and age, auxological measurements, pubertal stage, systolic and diastolic blood pressure, homeostasis model assessment-insulin resistance (HOMA-IR) index and lipid values were investigated.

**Results:**

A general decrease was observed in RNFL thickness in obese subjects compared to the controls, the decrease being highest in the inferior quadrant, although these differences were not statistically significant (p>0.05). RNFL thickness was negatively correlated with body mass index (BMI) standard deviation score (SDS) in both groups (control group r=-0.345, p=0.029; obese group r=-0.355, p=0.022). Significant negative correlations were determined between diastolic blood pressure, HOMA-IR, low density lipoprotein cholesterol level and RNFL thickness (r=-0.366, p=0.024; r=-0.394, p=0.016; and r=-0.374, p=0.022, respectively) in the obese group, while there was no association between these parameters and RNFL thickness in the control group.

**Conclusion:**

In this cross-sectional study, no statistically significant difference in RNFL thicknesses between the obese and control groups was determined. However, RNFL thickness was found to decrease in both healthy and obese children as BMI-SDS values increased. Further prospective studies may be of benefit to determine whether the decrease in RNFL values might become more pronounced on long-term follow-up.

## What is already known on this topic?

Obesity affects almost all systems in the body and can also cause injury to the retinal nerve fibers due to a chronic inflammatory process. The literature concerning this issue is scarce. Optical coherence tomography may show early retinal damage.

## 

### What this study adds?

Retinal nerve fiber layer thickness was found to be decreased in both obese and non-obese children as body mass index standard deviation score values increased.

## Introduction

The optic nerve carries signals originating from the retina to the visual cortex. Progressive loss of vision may occur when the transmission of these signals is impaired (1,2). The measurement of retinal nerve fiber layer (RNFL) thickness is a valuable tool for demonstrating early retinal damage. Optical coherence tomography (OCT) measures the delay in the reflection of laser light reflected from the retina. The RNFL can thus be visualized in a painless and non-invasive manner (3). Since it is rapid and simple, the technique is widely used in several diseases of the optic nerve and retina (optic neuropathies, retinal damage due to a range of causes, retinal vascular changes, glaucoma and so on). It is particularly useful in pediatric ophthalmology (4). Obesity is currently increasing globally. It affects almost all systems in the body and is a significant risk factor for vascular diseases (5,6). There are few studies in children and adolescents in this regard and they are controversial. The purpose of this study was to evaluate changes in RNFL thickness using OCT imaging in non-diabetic obese children and adolescents and to assess the association between any changes found and metabolic risk factors and pubertal stage. 

## Methods

This prospective observational study was performed with the approval of the University of Health Sciences Tepecik Training and Research Hospital Medical Research Ethical Committee (approval number: 29/12/2014-20) and in line with the ethical principles of the Declaration of Helsinki. 

Inclusion criteria for study and control subjects were;

1. Aged between 10-18 years old,

2. Not having neurological diseases,

3. Not having a history of ocular disease and/or surgery,

4. Children and their parents being compatible with the examinations,

5. Subjects with spheric values between -0.50 diopter (D)  and +0.50 D. 

Exclusion criteria for study and control subjects were;

1.Having diabetes mellitus or any systemic disease, 

2. Being on continuous medication, 

3. Not being sufficiently cooperative for OCT measurement. 

An ophthalmological assessment of both eyes was conducted on 38 obese children and adolescents aged 10.1-17.2 years who presented to the University of Health Sciences Tepecik Training and Research Hospital Clinic of Pediatric Endocrinology, İzmir, Turkey, between January 2015 and May 2016, and on 40 healthy children and adolescents aged 10.2-18.0 years who acted as the control group. Informed consent was obtained from patients and their families. Demographic data for the obese and control groups were obtained from their medical files. The control group was randomly selected from volunteers aged 10-18 years who were within normal limits for body mass index (BMI) standard deviation score (SDS) and in whom ophthalmological examination revealed no pathological findings. Anthropometric parameters, blood pressure values and pubertal stages were assessed by an experienced pediatric endocrinologist. Pubertal stages were classified according to Tanner and Whitehouse (7). Height measurements, accurate to the nearest centimeter, were performed using a rigid stadiometer. Weight measurement was accurate to the nearest 0.1 kg using a calibrated balance scale with the subject unclothed. Obesity was diagnosed according to World Health Organization criteria (8). BMI was determined using the formula weight (kg)/height (m^2^). Reference values established for Turkish children were employed to calculate SDS for weight, height and BMI (9). Blood pressure was measured in all cases after a period of resting and were repeated at least three times at 10 minute intervals. Subjects with systolic and/or diastolic blood pressure values greater than the 95^th^ percentile were regarded as hypertensive (10). Blood glucose, insulin and serum lipids in obese cases were measured using an automatic analyzer from fasting venous specimens taken on the same day. Insulin resistance using the homeostasis model assessment-insulin resistance (HOMA-IR) was calculated as fasting insulin (µIU/mL) × fasting glucose (mg/dL)/405 (11). All cases underwent comprehensive eye examinations by the same ophthalmologist. This included best corrected visual acuity, ocular motility examination and intraocular pressure using a Goldmann applanation tonometer, a detailed anterior segment assessment using a slit-lamp biomicroscope and optic nerve and retina examination using a 90 D lens. For pupil dilation, 1% cyclopentolate hydrochloride drops (Cycloplegin R; Abdi İbrahim İlaç Sanayi, İstanbul-Turkey) were used twice at 5 minute intervals, and the mean value was taken of three measurements performed 30 minute after the last drop using an auto-rephractometer device (Canon RK-F1). Subjects with spheric values between -0.50 D and +0.50 D were enrolled. RNFL thickness was measured using the OCT method (Spectralis HRA + OCT, 870 nm; Heidelberg Engineering, Heidelberg, Germany). In all cases, scans were carried out with pupillary dilatation under the identical level of dim room lighting by the same experienced technician. To avoid diurnal fluctuations, all OCT scans were performed at the same time in the morning. Internal fixation targets were employed in all tests together with a real-time eye tracking system in order to adjust for eye movements. RNFL thickness was determined around the disc with consecutive circular B scans (3.5 mm diameter). The thickness (between the interior margin of the internal limiting membrane and the exterior margin of the RNFL layer) was automatically segmented using the Spectralis version 6.3.2.0 software. Mean RNFL thicknesses were used in analyses. RNFL thickness measurements were taken from the optic disk and all surrounding quadrants of both eyes. Statistical analysis was performed with mean right and left eye RNFL values. Control and obese group RNFL values were then compared. Correlations between RNFL thickness and age, body measurements, pubertal stages, systolic and diastolic blood pressure, HOMA-IR and lipid values were investigated.

### Statistical Analysis

Statistical analysis was carried out using the Statistical Package for Social Sciences (SPSS 20.0; IBM, USA) software. The Kolmogorov-Smirnov test was used to assess the normality of the sample distribution. Mean and standard deviation values were given for all parameters. Partial correlations were used in relationship analysis for variables with normal distribution. Simple correlation analysis was applied to the variables for which normality was not provided. A p value <0.05 was considered statistically significant.

## Results

No differences were found between the study and control groups in terms of age, gender distribution, pubertal stages or systolic and diastolic blood pressure values (p>0.05). BMI-SDS values were 0.5±0.4 in the control group and 3.0±0.4 in the obese group (p<0.001). No difference was observed between the groups in terms of fasting blood glucose values (control group: 82.1±8.8 mg/dL, obese group: 85.3±9.9 mg/dL, p=0.65). Fasting insulin and HOMA-IR values were significantly higher in the obese subjects compared to the controls (fasting insulin: 19.6±9.8 vs. 8.3±3.1 mIU/mL, respectively, p=0.02; HOMA-IR: 4.7±2.7 vs. 1.9±0.7, respectively, p=0.01). There was no difference between the groups in terms of serum lipid levels (p>0.05). Clinical and laboratory characteristics of the groups are shown in Table 1. 

No difference between the sexes or between the two eyes was found at RNFL thickness evaluations using OCT imaging (p>0.05). RNFL thickness was lowest in both the control and obese groups in the nasal quadrant, followed by the temporal, superior and inferior quadrants in respective order. A general decrease in RNFL thickness was observed in obese subjects compared to controls, ranging from 2% to 7% in mean values, the greatest change occurring in the inferior quadrant, although these differences were not statistically significant. Obese and control group RNFL thickness measurements are shown in Table 2. Correlation analyses of RNFL with age, pubertal stage, BMI-SDS, blood pressure and metabolic parameters were performed. No correlation between RNFL thickness and age, pubertal stage, systolic blood pressure, fasting glucose, fasting insulin, triglyceride or high-density lipoprotein cholesterol were determined in the groups (p>0.05). RNFL thickness was negatively correlated with BMI-SDS in both groups (control group r=-0.345, p=0.029; obese group r=-0.355, p=0.022). Significant negative correlations were determined between diastolic blood pressure, HOMA-IR, low density lipoprotein-cholesterol level and RNFL thickness (r=-0.366, p=0.024; r=-0.394, p=0.016; and r=-0.374, p=0.022, respectively) in the obese group, while there was no association between these parameters in the control group. Correlations between clinical and laboratory values and RNFL thickness in the study and control groups are shown in Table 3.

## Discussion

Obesity and severe obesity have become a growing problem in children in recent years (5,6). The effect of obesity has been extensively investigated. However the effect of obesity on visual health, including RNFL, is one area in which there is a scarcity of data. RNFL thickness values in the children and adolescents with normal BMI SDS measured using OCT in this study were similar to those reported in the literature (12,13,14,15). Pehlivanoğlu et al (15) investigated RNFL thicknesses in healthy children and reported the thinnest values in the nasal and temporal quadrant and the thickest values in the inferior and superior quadrants. In agreement with that study, we also observed that the RNFL in both obese and control groups was thinnest in the nasal quadrant, followed by the temporal, superior and inferior quadrants in respective order. Pehlivanoğlu et al (15) reported the mean RNFL thickness values ​​of the right and left eyes in normal healthy Turkish children with a mean age of 10.7 years. However, measurements by age groups were not given separately in this study. The authors reported that the RNFL thickness did not change with age. In our study, RNFL thickness in 16 of the 38 cases (42.1%) in the obese group was below the normal values ​​reported by Pehlivanoğlu et al (15). In the control group, mean RNFL values ​​in 7 out of 40 (17.5%) patients were below the normal reference value. Clinical findings were not observed in any of the cases in which the RNFL thickness was lower than normal. Various studies have investigated the relationship between RNFL thickness values measured using OCT and variables such as age, sex and race. Budenz et al (16) reported a significant relationship between age and RNFL thickness, with a 2.2-µ decrease in RNFL thickness for every 10-year increase in age. El-Dairi et al (17) reported that RNFL thickness measurements in the under-18 years old population were not age-dependent. In our study in children and adolescents, RNFL thickness values ​​were not correlated with age. RNFL thickness values in the pediatric and adolescent age group can exhibit ethnic variation. Studies performed in the Turkish population show that mean RNFL thickness values are compatible with general values reported for Caucasians (18). The mean values in our study were also consistent with this. Although there was no statistically significant difference between the obese and control group RNFL values, a decrease was observed in all quadrants in the obese group compared to the controls, this change being greatest in the inferior quadrant. Similar studies have reported inconsistent results previously. In their study of obese children aged between 5 and 14, Pacheco-Cervera et al (2) reported a significant decrease in RNFL values in the severely obese group (BMI-SDS >4). A negative correlation was also determined in that study between RNFL values and serum leptin and interleukin (IL)-6 levels. In a study in adults, no correlation was reported between BMI and RNFL thickness in women, but a decrease was found in RNFL in men as BMI increased (19). Elía et al (20) found no relationship between BMI and RNFL thickness measured using OCT in healthy children. Karti et al (21) investigated 55 obese and 33 healthy children and reported a negative correlation between BMI-SDS and RNFL values. The presence of a refraction defect can cause inaccurate measurement of OCT and RNFL values (22,23). The study of Karti et al (21), included patients with high refractive status (up to 5 D), a factor which may have affected RNFL values by OCT. In our study, the refraction values ​​of all the cases that were recruited were between -0.5 and +0.5 D. We thus excluded any error caused by refraction defect from this study. There was no statistical difference in our study between the controls and the obese group in RNFL values despite a generalized reduction in RNFL in the obese subjects. However, correlation analysis revealed that RNFL thickness in both groups decreased as BMI-SDS values increased. The reason for the decrease in RNFL thickness in obese subjects is unclear. Pacheco-Cervera et al (2) suggested that RNFL values decreased with an increase in inflammatory mediators. Obesity is known to involve low levels of systemic inflammation (24,25,26). This long-term state of chronic inflammation may result in a decrease in RNFL values (27). It has been hypothesized that neuronal cell damage may occur in obesity due to changes in levels of hormones such as leptin and adipokines, and due to oxidative stress. Retinal ganglion cell (RGC) death occurs via apoptosis following axonal injury. The production of reactive oxygen species (ROS) is an important factor in RGC necrosis and apoptosis (21). Mac Nair et al (28) suggested that ROS can initiate RGC loss following axonal injury. This suggestion is supported by animal studies (29,30). Long-term chronic inflammation associated with obesity may cause a decrease in RNFL thickness through oxidative stress. Further studies are needed to clarify this hypothesis. Also there was a negative correlation between BMI-SDS and RNFL thickness in our healthy control group with normal BMI. In children with normal BMI, the increase in body fat may affect RNFL thickness although, again, the mechanism is unclear. Detailed longitudinal follow-up studies are needed on this issue.

### Study Limitations

As a limitation in this study, plasma levels of inflammatory mediators such as adiponectin, leptin and IL-6 were not measured. However, the effects of these adipokines in metabolic pathways were indirectly demonstrated by measuring insulin, glucose and lipid levels. The negative correlation between RNFL thickness and insulin resistance parameters supported the metabolic pathogenesis of retinal changes in obesity. No studies have shown whether changes may occur in RNFL values due to weight loss in obese subjects. Prospective observational studies involving weight control are needed to reveal the effect of obesity, and therefore of the chronic inflammatory process, on RNFL.

## Conclusion

In conclusion, in this study we observed a decrease in RNFL thickness in both healthy and obese children and adolescents as BMI-SDS values increased. No statistically significant differences in RNFL thickness between the obese and control groups were found. The decrease in RNFL values may have been revealed more clearly if the patients had been monitored prospectively. The clinical significance of the decrease in RNFL thickness is as yet unclear.

## Figures and Tables

**Table 1:**
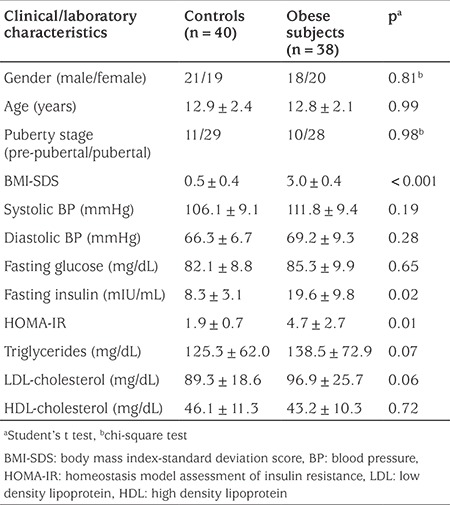
Clinical and laboratory characteristics of the study and control groups

**Table 2:**
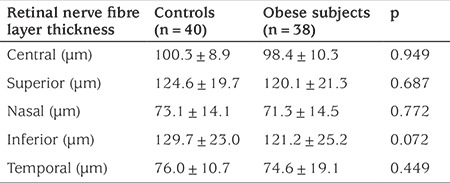
Mean ± standard deviation retinal nerve fiber layer thickness in controls and obese children

**Table 3:**
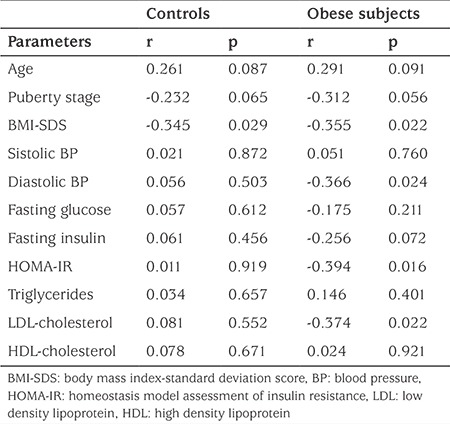
Correlation analysis of retinal nerve fiber layer with the clinical and laboratory parameters of the obese and control groups
